# Paternal bias expression of Igf2as is enhancer-dependent on the imprinting cluster of Igf2, H19 and Nctc1 in muscle cells

**DOI:** 10.1080/19768354.2019.1612780

**Published:** 2019-05-16

**Authors:** Jae-Young Kim, Hwa Young Youn, Karl Pfeifer, Bokkee Eun

**Affiliations:** a Department of Veterinary Internal Medicine, College of Veterinary Medicine, Seoul National University, Seoul, South Korea; b National Institute of Child Health and Development, National Institutes of Health, Bethesda, USA; c Core-Laboratory for Convergent Translational Research, Korea University College of Medicine, Seoul, South Korea

**Keywords:** Igf2as, Igf2, H19, imprinting, enhancer

## Abstract

Igf2, H19, and Nctc1 are linked co-regulated genes on distal mouse chromosome 7. This locus is an important model both for studying mechanisms of monoallelic expression and for elucidating the role of cis-regulatory elements – enhancers and insulators – in organizing chromatin and gene expression across a large domain. In this study we characterize regulated expression of the Igf2 antisense transcript (Igf2as) in primary muscle cells. We demonstrate that Igf2as is imprinted (expressed only from the paternal chromosome). We also show that Igf2as expression during differentiation follows the same patterns as Igf2 and H19. Moreover, this expression is dependent upon the same shared enhancer element. Thus, our work shows that the imprinted cluster includes Igf2as in addition to H19, Igf2, and Nctc1.

## Introduction

Genomic imprinting is a regulatory system in mammals where gene expression is determined by parental origin. There are at least 100 imprinted genes in the human genome and imprinting is widely conserved across mammalian species (Barlow and Bartolomei 2014). Imprinted genes are not scattered randomly across the genome but are organized into clusters of co-regulated genes. The H19/Igf2 imprinted gene cluster has been intensively studied as model to understand mechanisms for genomic imprinting. These studies have shown that epigenetic regulation of insulator/boundary elements via DNA methylation can alter promoter-enhancer looping interactions (Bartolomei and Tilghman 1997; Sha 2008). In addition to its importance as a model system for understanding imprinting mechanisms, the H19/Igf2 locus has direct biomedical relevance since loss of imprinting in this region is associated with developmental disorders (Beckwith Wiedemann and Russell-Silver syndromes) and with pediatric and adult cancers (Reik et al. 1996; Sun et al. 1997).

Many imprinting clusters contain long noncoding RNA (lncRNA) genes. At some imprinted loci, these lncRNAs are shown to be critical in establishing monoallelic gene expression. For example, transcription of Kcnq1ot1 and Air lncRNAs are essential for repression of Kcnq1 and Igf2R genes, respectively (Ideraabdullah et al. 2008). H19 itself is a lncRNA, but so far there is no evidence that H19 lncRNA helps establish genomic imprinting at the locus. Instead, the H19 lncRNA and/or the microRNA it encodes act in trans to regulate development (Cai and Cullen 2007; Kallen et al. 2013; Dey et al. 2014; Park et al. 2017). H19 and Igf2 are oppositely imprinted: H19 is transcribed only from the maternal chromosome while Igf2 is paternally transcribed. However, expression of each gene depends upon a series of shared tissue-specific enhancer elements located downstream of H19 (Leighton et al. 1995; Kaffer et al. 2001). The muscle specific enhancer is located at +26 kb upstream of the H19 transcription start site, within the intron 2 of the Nctc1 lncRNA gene. We have recently shown that Nctc1 lnRNA expression is dependent upon this shared Igf2/H19 enhancer element and that Nctc1 transcription is biased about 4:1 toward the paternal allele because the Nctc1 promoter must compete with H19 and with Igf2 for access to the shared enhancer. Curiously, we also saw that full activation of the muscle enhancer is dependent upon transcriptional activation of the Nctc1 promoter. Thus Nctc1 offers another example of a lncRNA playing an important role in controlling expression of an imprinted gene cluster. (Eun, Sampley, Good, et al. 2013; Eun, Sampley, Van Winkle, et al. 2013).

Igf2 antisense transcript (Igf2as) lies at the far distal end of the Igf2/H19 cluster, is highly expressed in embryonic tissues including skeletal muscle and cardiac muscle, and then its expression reduces gradually after birth (Rivkin et al. 1993; Duart-Garcia and Braunschweig 2014). Also, Igf2as transcription in the embryo, like Igf2 transcription, is paternally biased (Rivkin et al. 1993; Okutsu et al. 2000). However, the regulatory mechanisms controlling Igf2as transcription have not been directly studied. Especially, it is unclear whether Igf2as shares the skeletal muscle enhancer with H19, Igf2 and Nctc1.

In this study, we compared expression of Igf2as with H19, Igf2 and Nctc1 in primary myoblasts and in differentiating myotubes. We confirmed that expression of Igf2as is paternally biased and we show that expression is dependent upon the shared muscle enhancer element that drives expression of H19, Igf2, and Nctc1. Thus we redefine and thereby expand the extent of the H19/Igf2 imprinted cluster.

### Materials and methods

#### Animal studies

All mice were bred and housed in accordance with National Institutes of Health and United States Public Health Service policy. Animal research was approved through the Eunice Kennedy Shriver National Institute of Child Health and Human Development Animal Care and Use Committee.

Two kinds of wild type strains were used in this study. *FVB/NJ* (*Mus musculus domesticus*) was obtained from Jackson labs (Stock 001800) and *FVB/CAS* was generated as previously described (Gould and Pfeifer 1998). *FVB/CAS* is an *FVB/NJ* congenic but where the Igf2/H19 locus is *Mus musculus castaneus* in origin. Single nucleotide polymorphisms distinguish *castaneus* and *domesticus* alleles of Nctc1, H19, Igf2, and Igf2as. For simplicity, in the Results we refer to these two strains as *DOM* and *CAS*, respectively.

The *ΔME* allele carries a 26 kbp deletion that removes the shared muscle enhancer and the entire Nctc1 gene (Kaffer et al. 2001). The *ΔME* deletion is on a *domesticus* chromosome and thus carries SNPs equivalent to the wild type *DOM* strain.

#### Primary myoblast culture

Hind limb skeletal muscle was isolated from p3 to p5 pups. Myoblasts were isolated as described (Eun, Sampley, Van Winkle, et al. 2013) and grown at 37°C in 5% CO2 in F10 (Gibco) supplemented with 10% cosmic calf serum (Hyclone) and 5 ng/ml fibroblast growth factor (Peprotech). For differentiation, media was switched to DMEM supplemented with 5% horse serum.

#### Immunostaining

Cells were fixed in 4% paraformaldehyde for 10 min at room temperature and then blocked by treatment with 3% BSA and 0.2% Triton X-100 for 1 for 1 h at RT before incubation with antibodies. For MyoD (Abcam catalog No. ab16148) and MHC (DSHB catalog No. MF 20) staining, primary antibodies diluted 1:500 in blocking buffer were incubated at 4°C for 1 h and visualized with Alexa 546 diluted 1:1000 (Invitrogen A-21143 and A-11040, respectively). Nuclear staining was with DAPI (Molecular Probe). Images were composed and edited in Photoshop CS6 (Adobe).

#### RNA isolation and analysis

Hind limb skeletal muscle was isolated from neonates and RNA extracted using TRIZOL reagent (Thermo Scientific) according to the manufacturer's protocol. For cultured cells, total RNA was isolated with the RNeasy Plus Mini kit (Qiagen). For Northern blots, we used probes that were previously described (Kaffer et al. 2001; Eun, Sampley, Good, et al. 2013; Eun, Sampley, Van Winkle, et al. 2013). For qRT-PCR, cDNA samples were prepared with and without reverse transcriptase according using random hexamers (Transcriptor First Strand cDNA Synthesis Kit, Roche). cDNAs were analyzed using SYBR Green Master Mix I (Roche) on the Roche Cycler 480 (45 cycles with annealing at 60 °C) using primers described in Table 1. Expression was normalized to expression of Gapdh. Statistical significance was evaluated using Students *t-test*. Results in all figures show mean ± standard error. At least three independently derived primary cell lines were analyzed.

**Table 1.** List of primers.

Gene	Primer	Product (bp)
Igf2	5′-GAGCTTGTTGACACGCTTC-3′	357
	5′-ACGTTTGGCCTCTCTGAAC-3′	
Igf2as	5′-GGGAAGAAGTCACTACCTGAA-3′	268
	5′-AGTCAGTCACAGTATCTGGGA-3′	
Igf2as (for RFLP)	5′-CATGATAACAGAGCCCCTAGA-3′	360 (194/166: EcoNI)
	5′-CCAGGTGTCAATCCAGTGAAG-3′	
H19	5′-TGAGTTTCTAGGGAGGGAG-3′	532
	5′-ATTCCTGAGGCAGGTAGTG-3′	
H19 (for RFLP)	5′-GATGACAGGTGTGGTCAATGT-3′	330
	5′-CAGATTCCTGAGGCAGGTAGT-3′	
Nctc1	5′-AGATGAGCATGAAAGCCAAG-3′	242
	5′-TCCATCTCCCTTGCTGTATC-3′	
Gapdh	5′-CCTTCATTGACCTCAACTACAT-3′	399
	5′-CAAAGTTGTCATGGATGACC-3′	

##### Restriction fragment length polymorphism (RFLP) analysis

H19 and Igf2as PCR products were generated with primers described in Table 1 and digested with Cac8I or with EcoNI, respectively, to determine allelic origins. See https://phenome.jax.org/snp/retrievals.

## Results and discussion

### Primary myoblast cells are a good model for studying the role of the shared muscle enhancer.

**Figure 1.**
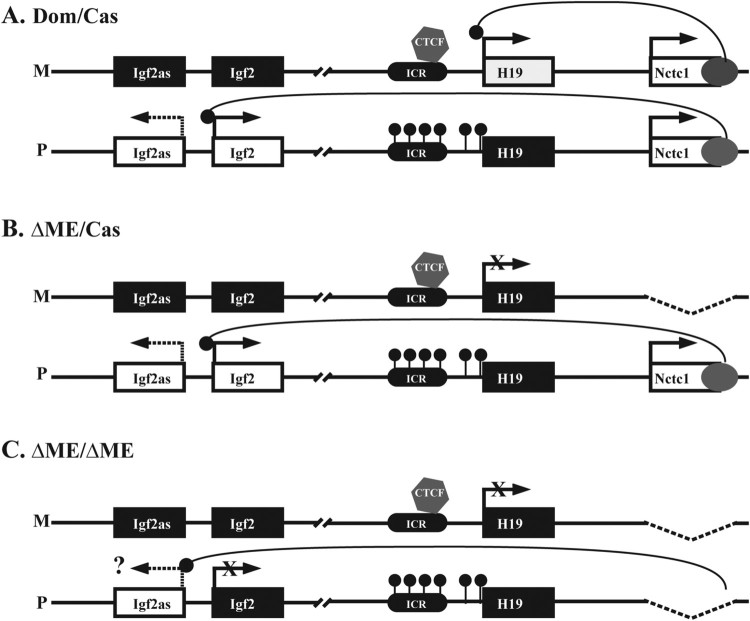
Summary of transcriptional regulation of the H19/Igf2 locus and depiction of alleles used in this study. (A) Regulation of imprinted expression at the wild-type locus. On the maternal chromosome, a CTCF-dependent insulator (or ICR, imprinting control region) (black oval) organizes the DNA loops between distal cis-regulatory elements to induce H19 expression and to prevent interactions between the Igf2 promoters and the shared downstream skeletal muscle enhancer (dark gray oval). Paternal-specific methylation of CpGs within the ICR prevents CTCF binding, resulting in Igf2 induction by allowing Igf2 promoter-enhancer loops. (B, C) Diagrams of the *ΔME* mutant alleles. The ΔME allele is a 26 kbp deletion of the shared muscle enhancer and Nctc1 gene. Expression of H19, Igf2, and Nctc1 have all been shown to be dependent upon this enhancer element (Kaffer et al. 2001; Eun, Sampley, Good, et al. 2013; Eun, Sampley, Van Winkle, et al. 2013). In this study we show that Igf2as expression is also dependent upon the shared enhancer. Rectangles denote the Igf2as, Igf2, H19, and Nctc1 genes, respectively. Small black oval denotes the ICR. The skeletal muscle enhancer is gray oval. Black rectangle means inactive genes and the white gene is actively transcribed. Black lollipops show the methylation of the CpG island on the paternal ICR.

To study regulation of Igf2as we generated wild type (*DOM/CAS*) pups along with littermates lacking the shared muscle enhancer on either the maternal chromosome *(ΔME/DOM*) or on both maternal and paternal chromosomes (*ΔME/ΔME*) (Figure 1). *DOM* and *CAS* are congenic wild type mice that differ only in regard to whether they carry single nucleotide polymorphisms (SNPs) from *Mus musculas domesticus* or from *Mus musculas castaneus* at the H19/Igf2 locus (Gould and Pfeifer 1998). The *ΔME* allele carries a 26 kbp deletion that removes the Nctc1 gene including the shared muscle enhancers (Kaffer et al. 2001). Note that the *ΔME* mutation is on a *domesticus* background.

**Figure 2.**
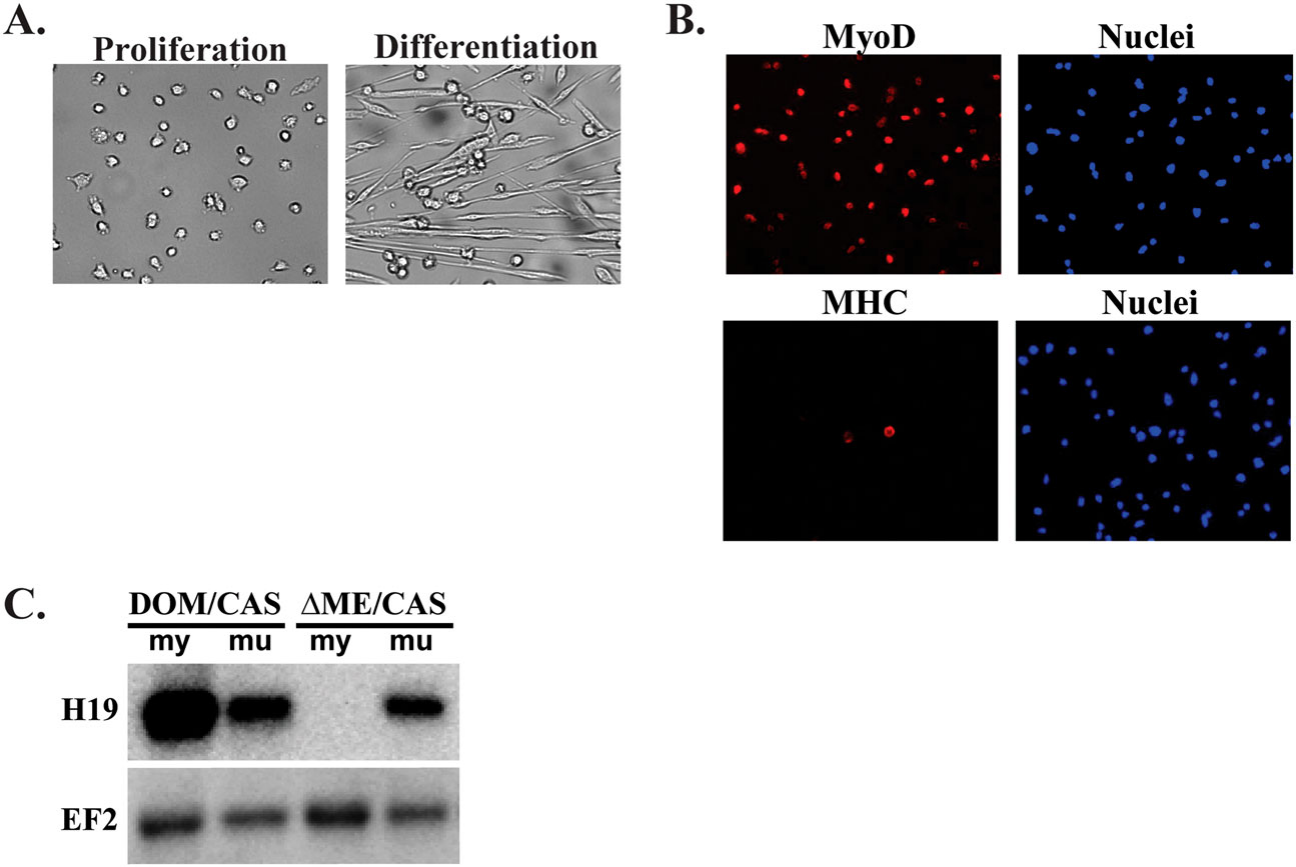
Characterization of primary skeletal myoblasts as a model for studying Igf2as gene regulation. (A) Primary myoblasts were derived from hind limb of wild type *DOM/CAS* pups at postnatal 3 days*.* Depicted as actively growing cells and cells after 48 h in differentiation media. (B) Immunostaining with myogenic and myocytic markers, MyoD and MHC. (C) (B) Northern blot analysis for H19 expression compares expression in muscle tissue or in primary cell lines derived from wild type (*DOM/CAS*) or from enhancer deletion *(ΔME/CAS*) littermates.

Primary myocyte cell lines were readily generated from wild type and from mutant pups. These cell lines grew robustly but upon serum depletion, cells differentiated efficiently into myotubes (Figure 2(A,B)).

A clear advantage to using primary cell lines is demonstrated in Figure 2(C) where we see that muscle enhancer ablation on the maternal chromosome results in complete abrogation of H19 expression in primary myotubes but reduces expression of H19 by only 3–4-fold in muscle tissue. Based on in situ hybridization (Kaffer et al. 2001) we know that residual H19 expression in tissue can be explained by high levels of H19 in fibroblasts and blood vessels that are unaffected by deletion of the muscle enhancer. Altogether, these data show that primary myocyte cultures offer a useful model for understanding mechanisms of gene expression during muscle cell differentiation and development. Therefore we proceeded to analyze Igf2as transcription in this model system.

#### Igf2as is imprinted paternal allele in the muscle cells

To determine if Igf2as is imprinted in muscle cells, we generated *DOM/CAS* and *CAS/DOM* pups, generated primary myocyte cultures, isolated RNAs from myoblasts and from myotubes, prepared cDNAs, and then performed restriction length polymorphism (RFLP) analyses.

**Figure 3.**
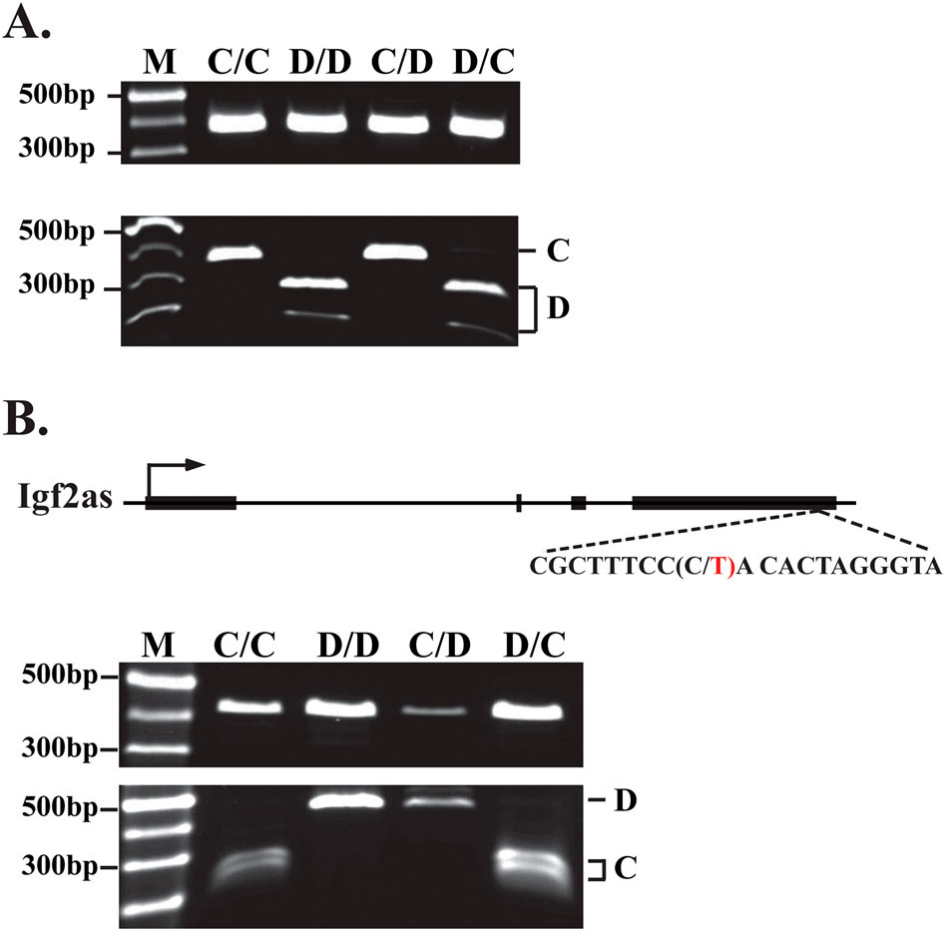
Igf2as is paternally imprinted. (A) RFLP analysis for H19 confirms that imprinting is maintained in primary cell line. RT-PCR products for H19 were digested with Cac8I enzyme to analyze presence or absence of distinguishing RFLP. (B) RFLP analysis for Igf2as. The cartoon drawing depicts the EcoNI polymorphism in the exon4 of Igf2as used to distinguish CAS and DOM cDNAs. RT-PCR products of Igf2as were digested with EcoNI and analyzed by gel electrophoresis on 1.5% agarose gels. In both panels A and B, C/C and D/D gDNA samples are included as controls. In both panels, undigested (top gel) and digested (bottom gel) samples are shown.

Figure 3(A) shows that H19 expression is only from the maternal chromosome. Similarly, Igf2 expression is only from the paternal chromosome (data not shown). These results demonstrate that our primary skeletal muscle cells maintain normal genomic imprinting.

To assay for Igf2as imprinting, we used an EcoN1 RFLP (Figure 3(B)). In *DOM/CAS* cell lines, Igf2as is always *castaneus* in origin. In *CAS/DOM* cell lines, Igf2as is always *domesticus*. Together these results demonstrate that Igf2as expression in myoblasts and myotubes is imprinted with expression exclusively from the paternal chromosome.

#### Expression patterns of Igf2as, Igf2, H19 and Nctc1 in differentiating myocytes/tubes.

**Figure 4.**
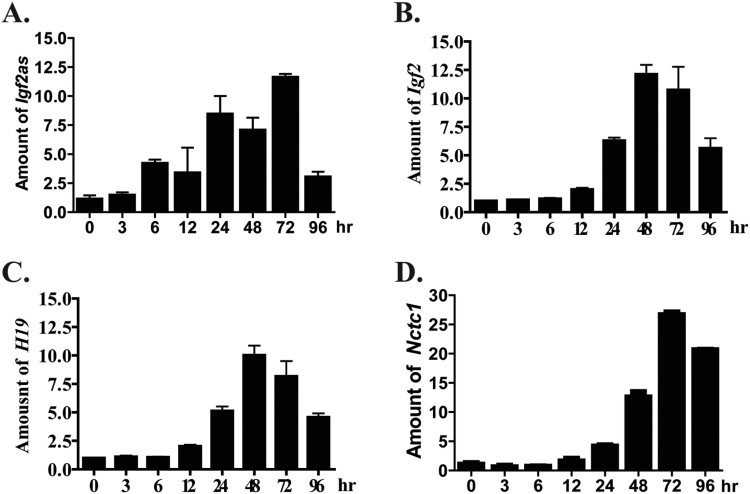
Differentiation dependent expression of Igf2as (A), Igf2 (B), H19 (C), and Nctc1 (D). Primary cell lines were derived from wild type (*DOM/CAS*) pups. Differentiation was induced by serum depletion and RNAs isolated at 0, 3, 6, 15, 24, 48, 72, and 96 h. For each gene, expression was normalized to Gapdh and then normalized to expression in cells before differentiation.

Igf2 and H19 are highly expressed in early development and in early postnatal stages. But they gradually decrease in tissues of adults (Duart-Garcia and Braunschweig 2014). We tested whether primary myoblasts/myotubes could provide a model to study temporal regulation of H19 and Igf2 expression and whether Igf2as shared in this temporal regulation. Specifically we performed qRT-PCR for Igf2as, Igf2, H19, and Nctc1 using RNAs prepared from myoblasts and from differentiating cells (Figure 4). The patterns are highly similar for all four genes, showing >10-fold induction upon differentiation that attenuates at about 72 h. The co-regulation of these genes is consistent with a common mechanism for regulating gene expression.

#### Igf2as is actively transcribed via sharing skeletal muscle enhancer.

**Figure 5.**
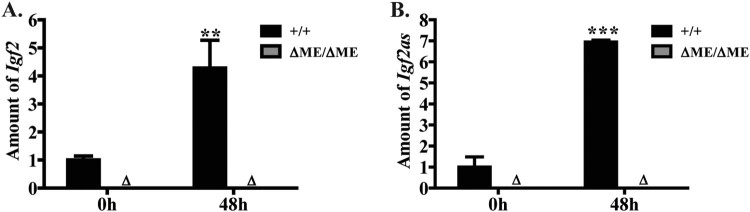
Transcription of Igf2as is entirely dependent upon the skeletal muscle enhancer. Primary cell lines were derived from wild type and from *ΔME/ΔME* littermates. Expression of Igf2 (A) and Igf2as (A) were analyzed by real-time RT-PCR using RNAs isolated from myoblasts and from differentiating cells (48 h). For each gene, expression was normalized to Gapdh and then normalized to expression of wild type cells before differentiation.

To directly test for a shared molecular mechanism for regulation of Igf2as with Igf2, H19, and Nctc1, we next assayed for Igf2as dependence upon the shared muscle enhancer. Specifically, we quantitated Igf2as RNA in wild type (*DOM/CAS*) myoblasts and myotubes in ΔME/ΔME myoblasts and myotubes. Just like Igf2 itself (Figure 5(A)), Igf2as expression is entirely dependent upon the upstream shared enhancer (Figure 5(B)).

In sum, we provide new evidence that Igf2as is an integral part of the H19/Igf2/Nctc1 imprinted cluster. Igf2as temporal expression patterns are indistinguishable from those of its neighbors. Moreover, its expression is absolutely dependent upon the shared muscle enhancer located about 90 kb upstream. Consistent with its dependence upon the shared enhancer and its location relative to the H19 Imprinting Control Region/Insulator element, expression of Igf2as is completely blocked on the maternal chromosome. It is interesting to note that Igf2as is in the opposite orientation relative to its cluster partners. Future studies will focus on the relevance of this orientation and the role of Igf2as in regulating expression of its neighbors.
